# Integrative multi-omics and systems bioinformatics in translational neuroscience: A data mining perspective

**DOI:** 10.1016/j.jpha.2023.06.011

**Published:** 2023-06-30

**Authors:** Lance M. O'Connor, Blake A. O'Connor, Su Bin Lim, Jialiu Zeng, Chih Hung Lo

**Affiliations:** a College of Biological Sciences, University of Minnesota, Minneapolis, MN, 55455, USA; b School of Pharmacy, University of Wisconsin, Madison, WI, 53705, USA; c Department of Biochemistry and Molecular Biology, Ajou University School of Medicine, Suwon, 16499, South Korea; d Lee Kong Chian School of Medicine, Nanyang Technological University, Singapore, 308232, Singapore

**Keywords:** Multi-omics integration, Systems bioinformatics, Data mining, Human brain profile reconstruction, Translational neuroscience

## Abstract

Bioinformatic analysis of large and complex omics datasets has become increasingly useful in modern day biology by providing a great depth of information, with its application to neuroscience termed neuroinformatics. Data mining of omics datasets has enabled the generation of new hypotheses based on differentially regulated biological molecules associated with disease mechanisms, which can be tested experimentally for improved diagnostic and therapeutic targeting of neurodegenerative diseases. Importantly, integrating multi-omics data using a systems bioinformatics approach will advance the understanding of the layered and interactive network of biological regulation that exchanges systemic knowledge to facilitate the development of a comprehensive human brain profile. In this review, we first summarize data mining studies utilizing datasets from the individual type of omics analysis, including epigenetics/epigenomics, transcriptomics, proteomics, metabolomics, lipidomics, and spatial omics, pertaining to Alzheimer's disease, Parkinson's disease, and multiple sclerosis. We then discuss multi-omics integration approaches, including independent biological integration and unsupervised integration methods, for more intuitive and informative interpretation of the biological data obtained across different omics layers. We further assess studies that integrate multi-omics in data mining which provide convoluted biological insights and offer proof-of-concept proposition towards systems bioinformatics in the reconstruction of brain networks. Finally, we recommend a combination of high dimensional bioinformatics analysis with experimental validation to achieve translational neuroscience applications including biomarker discovery, therapeutic development, and elucidation of disease mechanisms. We conclude by providing future perspectives and opportunities in applying integrative multi-omics and systems bioinformatics to achieve precision phenotyping of neurodegenerative diseases and towards personalized medicine.

## Highlights

• Significance of data mining of multi-omics datasets in translational neuroscience.
• Multi-omics data integration approaches: independent biological integration and unsupervised integration.
• Systems bioinformatics approach: towards the reconstruction of a comprehensive human brain profile.
• Applications in biomarker discovery, therapeutic development, and elucidation of pathogenic mechanisms of neurodegeneration.

## Graphical abstract

**Figure:**
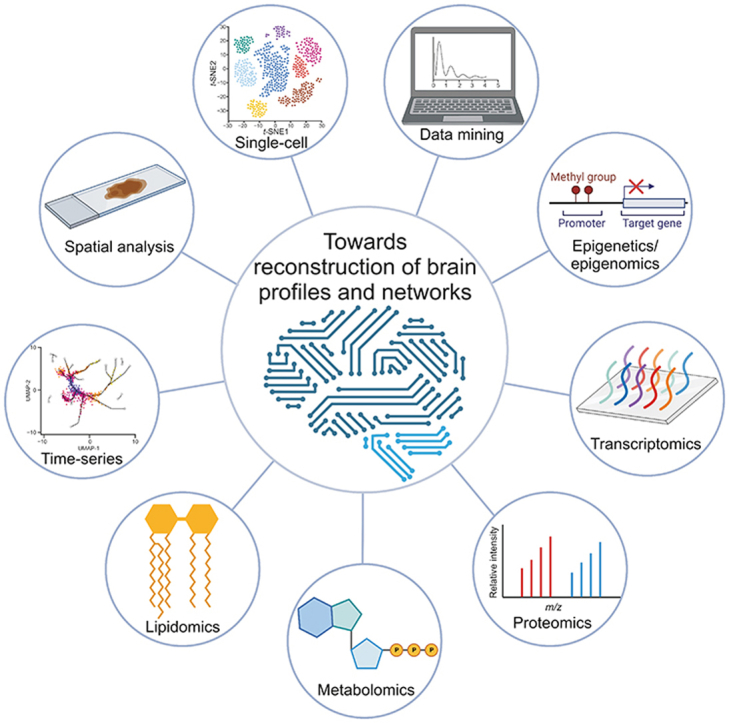

## 1 Introduction

The beginning of bioinformatics can be dated back to more than five decades ago, witnessing the parallel advances of computer science and experimental biology [1], including the advent of next-generation sequencing and omics technologies [2]. The omics technologies and analyses have been widely applied in neuroscience studies, ranging from the detection of alterations in genes (epigenetics/epigenomics), mRNA (transcriptomics), proteins (proteomics), and metabolites (metabolomics/lipidomics) at the molecular scale in the brain, their localization in different anatomical regions (spatial omics) to construct brain atlases, and the study of dynamics in biological processes by monitoring changes in individual cells (single-cell/single-nucleus trajectory inference) [[3], [4], [5], [6], [7], [8]]. Importantly, these omics studies provide a great depth of information and contain datasets that can be further analyzed and interpreted to support separate experimental observations or generate new hypotheses in neuroscience research [9].

Data mining of existing omics datasets to obtain novel biological insights opens new avenues to deepen the understanding of the clinical phenotypes, neuropathological features, disease progression, and pathogenic mechanisms of neurological disorders including Alzheimer's disease (AD), Parkinson's disease (PD) and multiple sclerosis (MS) [10]. In addition, data mining overcomes the technical challenges in conducting the initial experiments such as difficulty in obtaining precious tissue samples, stringent requirements in sample preparation, and high cost, while creating opportunities for new diagnostic and therapeutic strategies targeting neurodegenerative diseases. While single omics analyses have provided valuable disease mechanistic insights, recent studies have shown that integrative multi-omics analysis can help to define the connection and relationship among the different types of omics datasets to unravel brain networks regulating transitions from health to the development of neurological diseases and to classify clinically relevant subgroups to identify potential biomarkers [11,12].

**Fig. 1:**
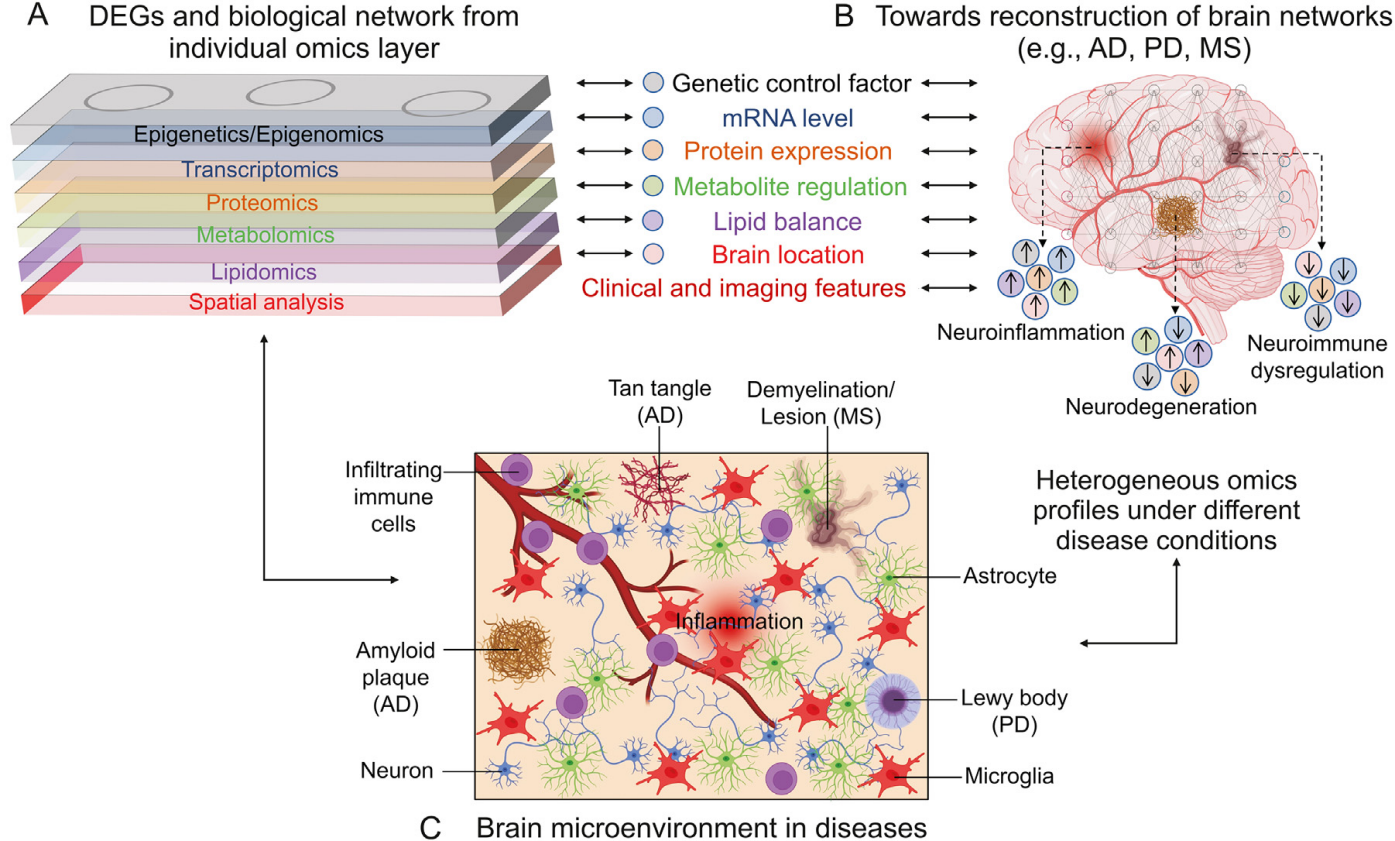
Towards reconstruction of brain networks and pathological profiles of neurodegenerative diseases by systems bioinformatics. (A) Integrated network of multi-omics data can be obtained from the individual omics layer. A combination of integrated networks with known clinical and imaging features may provide detailed molecular information towards reconstruction of neurodegenerative disease pathological profiles in the brain. (B) The presence of heterogeneous omics profiles under various disease conditions, such as neuroinflammation, neurodegeneration, and neuroimmune dysregulation, which can be characterized by different combinations of alterations in the omics layers. (C) The reconstructed brain networks could reflect the pathological profiles associated with the true biological systems and the microenvironments under specific disease states. The figure was created with BioRender.com. DEGs: differentially expressed genes; AD: Alzheimer's disease; PD: Parkinson's disease; MS: multiple sclerosis.

It is hence crucial to understand the neurodegenerative pathology from the perspective of individual type of omics data obtained from the human samples, followed by adopting the systems bioinformatics approach to integrate the multiscale and multisource big data from each omics layer to allow for a holistic analysis of the complex brain system (Fig. 1A). Importantly, there is heterogeneous omics profiles under various disease conditions, such as neuroinflammation, neurodegeneration, and neuroimmune dysregulation, which can be characterized by different combinations of alterations in the omics layers (Fig. 1B). In addition, further development and optimization of muti-omics integration approaches and pipelines will potentially enable the reconstruction of comprehensive brain networks and pathological profiles reflective of the biological systems and the microenvironments under specific disease states (Fig. 1C) [5,[13], [14], [15], [16]]. Furthermore, there is a need for thorough interpretation of the outcomes from data mining and their relevance to the true biological observations.

In this review, we first summarize data mining based translational neuroscience studies that performed secondary bioinformatics analysis utilizing deposited datasets from a spectrum of omics technologies including epigenetics, transcriptomics, proteomics, metabolomics, lipidomics, and spatial omics. We then discuss current methods of multi-omics integration and propose a systems bioinformatics approach to work towards the reconstruction of brain networks and pathological profiles for increased accuracy and reliability in recapitulating the true biological systems of the brain. We further recommend combining high dimensional bioinformatics analysis with experimental validation in biomarker discovery, therapeutic development, and elucidation of disease mechanisms. We conclude by providing future perspectives and opportunities in utilizing integrative multi-omics and systems bioinformatics to achieve targeted therapies for neurodegenerative diseases and advance towards personalized medicine.

## 2 Omics spectrum-based data mining in translational neuroscience

Many factors contribute to neurodegeneration, including but not limited to, pathogenic mutations leading to mutant protein production and toxic protein aggregation [[17], [18], [19], [20]], as well as altered cytokine production, and dysregulated cellular signaling pathways [21,22]. As opposed to conventional interventions aimed at specific toxic protein or aberrant receptor signaling, interventions at the gene level can more effectively reduce the negative downstream cellular pathogenic effects that arise from malfunctioning of a single protein. Gene targeting strategies can be particularly beneficial in neurodegenerative diseases that result from the toxic gain-of-function of a protein where a significant loss of its normal function does not have adverse effects on the cells [23]. Furthermore, there is a need for better understanding of the regional effects of a gene or protein in the brain as well as the time-dependent changes in their expression or functions to enable specific targeting and more effective treatments. In this section, we will summarize the individual omics-based data mining studies in translational neuroscience and their applications in guiding biomarker and therapeutic development.

### 2.1 Epigenetics/epigenomics analysis

With recent advancements in experimental and computational tools to analyze neurodegenerative diseases, a genetic basis for these diseases has yet to be fully elucidated [24]. In this section, we will discuss the gene-centric view of understanding the role of epigenetics/epigenomics in the pathogenesis of neurodegenerative diseases. Epigenetics can be summarized as heritable changes in gene function which are not encoded by nucleotide sequences in DNA, but influence gene expression and subsequent protein expression levels without altering the DNA sequence [25,26]. These changes can be due to major epigenetic mechanisms such as DNA methylation, histone modifications, chromatin remodeling, non-coding RNA regulation, as well as environmental factors such as diet, and exposure to chemicals which are dynamic and reversible [[24], [25], [26], [27]], making them viable targets for therapeutic developments [28]. Experimental studies exploring the role of epigenetic mechanisms in neurodegenerative diseases have found that DNA methylation variations may affect beta-amyloid (Aβ) and tau deposition in AD, and expression of α-synuclein in PD [29,30]. While epigenetics/epigenomics datasets are deposited in various databases, including DeepBlue [31], EWAS Open Platform [32], Genomic Expression Archive (GEA) [33], Genome Wide Associated Studies (GWAS) [34], IHEC Data Portal [35], National Cell Repository for Alzheimer's disease (NCRAD) [36], Roadmap Epigenomics [37], and Gene Expression Omnibus (GEO) [38] (Table 1, Epigenetics/Epigenomics database), there are limited number of data mining studies.

**Table 1:** List of databases and repositories that are available for data mining of different types of omics datasets.

Databases and repositories	Data types	Refs.
DeepBlue Epigenomic Data Server	Epigenetics/Epigenomics	[31]
Epigenome-Wide Association Study (EWAS) Open Platform	Epigenetics/Epigenomics	[32]
Genomic Expression Archive (GEA)	Epigenetics/Epigenomics	[33]
Genome Wide Associated Studies (GWAS) Catalog	Epigenetics/Epigenomics	[34]
International Human Epigenome Consortium (IHEC) Portal	Epigenetics/Epigenomics	[35]
National Cell Repository for Alzheimer's Disease (NCRAD)	Epigenetics/Epigenomics	[36]
Roadmap Epigenomics	Epigenetics/Epigenomics	[37]
Gene Expression Omnibus (GEO) Database	Epigenetics/Epigenomics/Bulk RNA-seq	[38]
Mount Sinai Brain Bank	Bulk RNA-seq	[54]
ROSMAP Database	Bulk RNA-seq	[55]
ARCHS4 Database	Bulk RNA-seq/sc/snRNA-seq	[56]
DDBJ Sequence Read Archive (SRA)	Bulk RNA-seq/sc/snRNA-seq	[57]
Synapse Database	Bulk RNA-seq/sc/snRNA-seq	[58]
scREAD Database	sc/snRNA-seq	[69]
Allen Brain Map	sc/snRNA-seq	[70]
DRscDB Database	sc/snRNA-seq	[71]
Global Proteome Machine Database (GPMDB)	Proteomics	[87]
jPOSTrepo (Japan ProteOme STandard Repository)	Proteomics	[88]
MassIVE	Proteomics	[89]
Proteomic Data Commons	Proteomics	[90]
ProteomeXchange	Proteomics	[91]
PRoteomics IDEntifications Database (PRIDE)	Proteomics/Spatial Omics	[92]
ProteomicsDB	Proteomics/Spatial Omics	[93]
Alzheimer's Disease Neuroimaging Initiative (ADNI)	Metabolomics/Lipidomics	[110]
Cerebrospinal Fluid Metabolome Database	Metabolomics/Lipidomics	[111]
Human Metabolome Database	Metabolomics/Lipidomics	[112]
Lipid Bank	Metabolomics/Lipidomics	[117]
LipidBlast	Metabolomics/Lipidomics	[118]
Lipid MAPS	Metabolomics/Lipidomics	[119]
MetaboAge Database	Metabolomics/Lipidomics	[120]
MetaboLights	Metabolomics/Lipidomics	[121]
Metabolomics Workbench	Metabolomics/Lipidomics	[122]
MetabolomeXchange	Metabolomics/Lipidomics	[123]
Serum Metabolome Database	Metabolomics/Lipidomics	[124]
Omics Discovery Index (OmicsDI)	All Omics	[127]
Dynamic Proteomics	Spatial Omics	[165]
Giotto	Spatial Omics	[166]
Spatial TranscriptOmics DataBase (STOmicsDB)	Spatial Omics	[167]
SpatialDB	Spatial Omics	[168]

This has led to the need to overcome practical impediments including developing algorithms and models for large-scale data mining of epigenetics/epigenomics datasets [39]. One data mining study utilizing DNA methylation data obtained from the GEO database implemented a supervised machine learning algorithm, including the construction of differential network related to aging acceleration and the use of Markov Chain Monte Carlo method of global sensitivity analysis, to better understand the accelerated epigenetic aging mechanisms of various neurodegenerative diseases [40]. Their results indicated that individuals with neurodegenerative diseases exhibited a significantly accelerated aging pattern. Specifically, they found that *CDCA7L* and *EFNB2* are significantly different than other genes in AD and PD, respectively. While *CDCA7L* is involved in neuronal death, and *EFNB2* is involved in apoptosis and the development of the nervous system as well as neuronal migration. Their analysis further revealed that *DUSP1*2 had the largest betweenness across different disease types, and that *DUSP12* may regulate the c-Jun N-terminal kinase signaling pathway by dephosphorylating its substrate, which is critical to cell differentiation, apoptosis, and other neural functions in the progression of neurodegenerative diseases [40].

Another data mining study implemented a new computational framework, including the use of the DBSCAN algorithm and Limma statistical methods, to analyze GEO datasets and identified 21 and 89 differentially methylated genes for AD and Down syndrome respectively [41]. Their evaluation indicated high classification accuracy of these two methylation signatures with 92% for AD and 70% for Down syndrome. Their framework is capable of detecting outlier-free epigenetic signatures in complex diseases, with applications to analyze various epigenetic signatures throughout disease pathogenesis [41]. Studies performing meta-analyses of epigenomic datasets have found differentially methylated genes in varying brain regions [41], as well as age-associated methylation patterns concurrent with epigenetic dysregulation observed in AD [42].

It is important to note that epigenetic features and RNA expression as well as the subsequent protein expression level do not necessarily have a direct correlation [43]. With most drugs targeting proteins, it is vital to take a systems bioinformatics approach and integrate multiple types of omics data to holistically understand the disease mechanisms and regulation of protein synthesis at all levels of the central dogma [44]. There is also a need to characterize epigenetic changes at the cell-specific level with spatial resolution [[45], [46], [47], [48], [49]]. Going forward, personalized medicine aimed at targeting specific epigenetic changes in patients with neurodegenerative diseases is poised to drastically transform diagnostic and therapeutic strategies [50].

### 2.2 Bulk and single-cell/single-nucleus RNA sequencing analysis

One of the most commonly used methods in transcriptomics for gaining insight into which genes are differentially expressed in varying disease states is the RNA sequencing (RNA-seq). Transcriptomics analysis has been used to produce data representative of mRNA expression levels of tens of thousands of genes, as well as the identification of the differentially expressed genes (DEGs) in various biological samples between patients and healthy controls. RNA-seq allows for the sequencing of the whole transcriptome [51] and provides additional information on splice variants or non-coding RNA [52,53]. The databases that archive RNA-seq datasets include GEO [38], Mount Sinai Brain Bank [54], ROSMAP database [55], ARCHS4 [56], DDBJ Sequence Read Archive (SRA) [57] and Synapse database [58] (Table 1, Bulk RNA-seq database).

A data mining study focusing on biomarker discovery for AD utilized RNA-seq datasets stored in the DDBJ SRA and identified the gene *NEUROD6* to be downregulated in the brain tissue of AD patients [59]. Another study utilizing RNA-seq datasets obtained from the ROSMAP database and Mayo Clinic studies found that disease pseudotime (an arbitrary unit of time to measure a cells progression) in AD is significantly concordant with the burden of tau, Aβ, and cognitive diagnosis of late-onset AD [60]. Additionally, it was reported that early-stage disease pseudotime samples show changes in basic cellular functions, while the late stage disease pseudotime samples show changes in neuroinflammation and amyloid pathologic processes [60].

Another data mining study uses the DDBJ SRA to obtain RNA-seq datasets from 26 different studies involving brain tissues and blood samples of AD and PD patients for meta-analysis [61]. By applying a random forest-based machine learning algorithm to analyze existing central and peripheral transcriptomic data, it was found that there is little overlap between AD and PD. Interestingly, the study revealed an overlap between central and peripheral transcriptomic signatures in PD that are characterized by anomalies in exocytosis and specific genes related to the SNARE complex including vesicle-associated membrane protein 2 (*VAMP2*), syntaxin 1A (*STX1A*), and p21-activated kinase 1 (*PAK1*) [61]. In a separate PD study making use of RNA-seq datasets from the GEO database, several genes including *RPL21*, *RPL34*, *CKS2*, *B2M*, *TNFRSF10A*, *DTX2*, and *HLA-B*, have been shown to be upregulated in PD brain tissues [62]. In MS, a data mining study analyzing RNA-seq datasets from the GEO database has reported that inactive MS brain lesions contain significantly more M2 macrophages compared to normal white matter controls [63].

Besides bulk RNA-seq, studies using single-cell RNA-seq (scRNA-seq) and single-nucleus RNA-seq (snRNA-seq) techniques are of high interest because they provide not only the average expression level for an ensemble of cells such as in the typical RNA-seq analysis [64,65], but also the ability to quantify gene expression levels in specific cell types [[64], [65], [66], [67], [68]]. Both techniques provide greater depth and insight into the analyzed data when compared to bulk RNA-seq. The scRNA-seq and snRNA-seq approaches are important in revealing cell subpopulations and intercellular heterogeneity, understanding regulatory relationships between genes, and tracking trajectories of distinct cell lineages in development, to understand disease pathogenesis [64,65]. In particular, the ability of scRNA-seq and snRNA-seq techniques to dissect the functional changes of highly heterogeneous cells in the brain at the single-cell level can significantly improve our understanding of the vulnerability of particular cell types in certain neurodegenerative diseases [69]. Some of the common databases and repositories used for data mining of scRNA-seq/snRNA-seq datasets include ARCHS4 [56], DDBJ SRA [57], Synapse database [58], Allen Brain Map [70], DRscDB database [71], and scREAD database [69] (Table 1, sc/snRNA-seq database).

A study making use of scRNA-seq datasets from scREAD to analyze the entorhinal cortex of AD brains found that phosphoinositide 3‑kinase (PI3K)/protein kinase B (AKT) signaling, Wnt signaling, neuroactive ligand-receptor interaction pathways, and neurodegeneration pathways were significantly impaired in astrocytes from the entorhinal cortex of AD patients [72]. A similar data mining study using scRNA-seq data in combination with bulk RNA-seq data obtained from the Synapse database found significant upregulation of *PLCG2* expression in AD patients which positively correlates with amyloid plaque density [73]. This finding was validated by using an AD mouse model which showed increased *PLCG2* expression associated with amyloid pathology and disease progression and reducing microglia reverses the disease pathology [73]. A different study analyzed three snRNA-seq datasets [[74], [75], [76]] and showed that *LINGO1* is upregulated in both excitatory neurons and oligodendrocytes, together with indication of mitochondrial and estrogen signalling dysfunction in AD [77].

Finally, a study integrated both scRNA-seq datasets from scREAD and bulk RNA-seq datasets obtained from the Mount Sinai Brain Bank and ROSMAP databases for a comprehensive drug repositioning analysis [78]. They identified multiple new candidates for AD treatment such as trichostatin, which was predicted to be broadly applicable to different AD subtypes, and vorinostat, which was specific for one subtype of AD, and both of which are histone deacetylase inhibitors [78]. It is important to note the lack of scRNA-seq and snRNA-seq data mining studies for PD and MS which might be due to the limited number of databases dedicated to compiling scRNA-seq/snRNA-seq data for these diseases. While providing greater depth, scRNA-seq and snRNA-seq are lacking the spatial information which is achievable with image-based transcriptomics which we will discuss in the subsequent section.

### 2.3 Proteomics analysis

Functional proteins and their interactions with other molecules are essential for biological processes and cellular functions which govern the disease mechanisms in neurodegenerative diseases [79]. Importantly, translation of proteins from RNA is not a linear relationship, where some genes may not even translate into functional proteins [[80], [81], [82]]. Therefore, it is important to characterize protein expression in addition to RNA quantification when investigating disease states [79]. Proteomics provides additional biological insights such as protein-abundance differences in proteomes, time-dependent expression patterns, post-translational modifications, and protein-protein interactions (PPIs) that otherwise could not be obtained from transcriptomics [79]. These parameters have been shown to have paramount effects on the pathogenesis and progression of neurodegenerative diseases [83].

Proteins rarely act as isolated machinery, and rather, their functionality is highly related to the proteins they interact with. Therefore, proteins whose function is well understood may be used to predict the function of unidentified proteins [84]. Both experimental and computational studies of PPI has enabled and expedited the modelling of functional pathways to elucidate the pathogenic mechanisms of cellular processes and identify their translational applications [85,86]. There is currently a lack of data mining of proteomics studies, although there have been efforts to compile mass spectrometry proteomics datasets into databases such as Global Proteome Machine Database (GPMDB) [87], jPOSTrepo [88], MassIVE [89], Proteomic Data Commons [90], ProteomeXchange [91], PRoteomics IDEntifications Database (PRIDE) [92], and ProteomicsDB [93] (Table 1, Proteomics database). ProteomicsDB is an example of a database that allows users to explore and retrieve protein abundance values across different tissues, cell lines, and body fluids [93].

A neurodegenerative disease focused data mining study compiled proteomics studies of post-mortem brain tissue and used a meta-analysis approach to discover that biological processes related to the organization of the extracellular matrix, metabolism of glycosaminoglycans and proteoglycans, blood coagulation, response to injury, and oxidative stress were highly dysregulated in AD, PD, and Huntington's disease through PPI network analysis and Gene Ontology (GO) enrichment analysis [94]. Another study utilized the PRIDE database to study post-translational modifications in AD patients. They report 103 proteins with post translational modifications that are uniquely expressed between brain region with no tangles, intermediate tangles, and severe tangles [95]. The bioinformatics analysis suggested the association of these proteins in AD progression through platelet activation, and they were found to be enriched for the tricarboxylic acid cycle (Kreb's Cycle), respiratory electron cycle, and detoxification of reactive oxygen species [95]. Another proteomics study making use of meta-analysis found that pathways related to synaptic signaling, oxidative phosphorylation, immune response, and extracellular matrix were commonly dysregulated in AD through bioinformatic gene set enrichment analysis with the Enrichr web server [96,97].

Proteomic expression data provides insight into the involvement of post-transcriptional editing and quantifies protein encoding mRNA genes that make it through translation, and ultimately play critical roles as functional proteins [80,81,98]. As bioinformatics analysis is becoming more refined and expansive, it is vital for technological advances to keep up with the growing need for specificity. The importance of using combinatorial methods of transcriptomics and proteomics is now well established [99,100]. Single-cell proteomics analysis techniques are now bridging the gap by filling the growing need of specificity in understanding cell heterogeneity in neurodegenerative disease tissues [99]. Single-cell proteomics makes use of mass spectrometry techniques for proteome quantification by analyzing individual cells one at a time [99,100]. This new and growing field will give novel insights into cell heterogeneity in tissue samples, as well as providing information into transcriptional regulation of DEGs when compared to scRNA-seq datasets [82,99,101].

### 2.4 Metabolomics/lipidomics analysis

Metabolomics and lipidomics were once classified under the same umbrella, but each of them now occupies an independent domain due to the large range of studies characterized by each of the methods extensively [102]. Metabolomics focuses mostly on examining polar metabolites such as sugars, amino acids, organic acids, and nucleotides which are usually the end products of complex biochemical cascades. On the other hand, lipidomics strives to identify lipid molecular species which should be analyzed separately from small-molecule metabolites due to their hydrophobic nature [103,104]. Importantly, with the brain being the second most abundant organ in terms of lipid concentration and diversity, lipid dysregulation has been largely linked to AD, PD, and MS due to the vital tasks of lipids in myelination of neurons and signal transduction via lipid mediators [105].

Metabolomics and lipidomics datasets are typically acquired from similar biological samples using common analysis methods such as mass spectrometry, ion chromatography, liquid chromatography, and nuclear magnetic resonance [103,106,107]. Data collected from metabolomics and proteomics are not mutually exclusive to each other, due to the intertwined relationship they both have to biological processes involved in cellular homeostasis and pathogenesis of neurodegenerative diseases, which will contribute to potential diagnosis and therapeutic targeting of these diseases [108,109]. With the expanding experimental data being collected, there are now many openly accessible metabolomics and lipidomics databases such as Alzheimer's Disease Neuroimaging Initiative (ADNI) database [110], Cerebrospinal Fluid (CSF) Metabolome Database [111], Human Metabolome Database (HMDB) [[112], [113], [114], [115], [116]], Lipid Bank [117], LipidBlast [118], Lipid MAPS [119], MetaboAge Database [120], MetaboLights [121], Metabolomics Workbench [122], MetabolomeXchange [123], and Serum Metabolome Database [124] (Table 1, Metabolomics/Lipidomics database).

In terms of lipidomics, a data mining study utilized datasets obtained from the ADNI database [110], which consisted of 349 serum samples obtained form 806 participants, to investigate lipid metabolism in AD [125]. They found lipid desaturation, elongation, and acyl chain remodeling processes to be disturbed in the blood of AD patients. The study further tested the association between sets of blood lipids with known AD biomarkers and showed that Aβ in the CSF correlates with glucosylceramides, lysophosphatidylcholines, and unsaturated triacylglycerides [125]. On the other hand, there is a scarcity of metabolomics-based data mining studies associated with neurodegenerative diseases. To investigate aging and associated diseases, MetaboAge database has compiled metabolomics data from dozens of studies reporting statistically significant changes in metabolites associated with ageing in healthy individuals [120]. This database may serve as an informative platform to compare metabolic changes between ageing and the mechanisms of neurodegenerative diseases obtained from other databases to facilitate future data mining studies using metabolomics datasets.

As examples, there are several data mining studies utilizing metabolomics datasets to understand the changes in metabolites in other neurological diseases such as glaucoma and depression. A meta-analysis study looking at primary open angle glaucoma (POAG) identified aminoacyl-tRNA biosynthesis and arginine metabolisms, which play important roles in immune responses, being dysregulated in patients with POAG compared to controls [126]. Another study examining altered metabolites in depression compiled 5,675 metabolite entries from 464 studies collected from metabolomic databases, including HMDB, MetaboLights [121], Metabolomics Workbench [122], MetabolomeXchange [123], as well as Omics Discovery Index [127] which contain all omics datasets, together with extensive literature survey [128]. They found that patients with depression had lower levels of brain gamma-aminobutyric acid and glutamate/glutamine, and that tryptophan metabolism-related metabolites such as serotonin, 5-hydroxyindoleacetic acid, quinolinic acid, and tryptophan were most frequently changed after treatment [128].

### 2.5 Spatial omics analysis

Spatial omics technologies have provided new opportunities to visualize the anatomical localization of biological molecules to enable the investigation of the structural organization of complex tissue as well as visualization of the interactions between cells and their tissue microenvironments [[129], [130], [131], [132]]. While spatial analysis has been increasingly applied to all types of omics studies, most of the current studies focus on spatial transcriptomics and proteomics analyses. A number of technological advances have enabled transcriptomics and proteomics profiling where the transcripts or proteins can be assigned to their specific cell types and cell location [[133], [134], [135], [136]], revealing distinct spatial patterns of cells in tissues that were previously inferred through indirect means [[137], [138], [139]]. Techniques used for spatial transcriptomics analysis include fluorescence in situ hybridization (FISH) [140], seqFISH+ [136], and multiplexed error-robust FISH (MERFISH) [133], while spatial proteomics analysis makes use of techniques such as cytometry by time of flight (CyTOF) [141] and highly multiplexed immunofluorescence imaging approaches [142]. All analytical techniques have been applied to analyze AD [76,[143], [144], [145]], PD [[146], [147], [148]], and MS tissues [[149], [150], [151], [152], [153]].

To facilitate the interpretation of spatial transcriptomics and proteomics datasets, interactive visualization tools are typically used, including SpatialLIBD [154], SpatialExperiment [155], Bento [156], MSnbase [157], pRoloc [157], Squidpy [158], Spatial Multi-Omics (SM-Omics) [159], ATHENA [160], and TRANSPIRE [161,162]. These analysis tools process single-cell transcriptomics and proteomics data and computes spatial statistics of subcellular RNA and protein molecular distributions, compartmental expression, and cell morphology to build multidimensional biological features associated with diseased states as compared to controls [156]. Databases and repositories can further facilitate the comprehensive archiving and exploration of spatial omics datasets to enable data mining or comparisons with other experimental data [163,164]. Commonly used databases include PRIDE [92], ProteomicsDB [93], Dynamic Proteomics [165], Giotto [166], Spatial TranscriptOmics DataBase (STOmicsDB) [167], and SpatialDB [168] (Table 1, Spatial Omics database). For example, SpatialDB contains functions such as the ability to search for relevant publications and tools, public dataset visualization, customized specialized databases, new data archive, and online analysis [168]. It is important to note that while there are several analytical tools and databases available to aid in spatial transcriptomic and proteomics data analysis, not many studies have made use of such tools for data mining.

A prominent example is Giotto, which utilizes a rich variety of algorithms that enables robust spatial data analysis, and a user-friendly platform for data visualization and exploration, including characterizations of tissue composition, spatial expression patterns, and cellular interactions [166]. Giotto has been shown to be applicable to a wide range of public datasets, including several spatial datasets from neurodevelopment and neurodegeneration studies that illustrated consistent analysis and conclusion [166]. In addition, RNA-seq data can be integrated for spatial cell-type enrichment analysis [166,169,170]. Giotto, for example, has utilized single-cell spatial transcriptomic MERFISH data that was collected from the pre-optic cortex of a mouse, and was able to identify 8 distinct cell clusters as well as creating interactive three-dimensional plots of the dataset [166] where the results are concordant with the original study from which the data was obtained. Giotto has additionally been used to predict the presence of a given cell type in a spatial location with multiple cell types for datasets with low spatial resolution. The spatial cell type prediction algorithm was tested by altering a seqFISH + dataset to mimic low spatial resolution. The cell-type enrichment analysis was conducted by using scRNA-seq data and derived marker gene lists for somatosensory cortex associated cell types obtained from a previous study [171], where there is a high accuracy in predicting the presence of a cell type at each individual spatial location [166].

A data mining study made use of a multi-modal structured embedding (MUSE) approach to analyze five datasets consisting of seqFISH+, STARmap, spatial transcriptomics, Visium, and spatial transcriptomics with fluorescent imaging data [172]. Application of MUSE to these diverse datasets yielded spatial patterning in healthy mice brain cortex tissue utilizing seqFISH + data, as well as heterogeneity of amyloid precursor protein processing in mice AD brain regions [172]. MUSE also successfully clustered STARmap mouse cortex data and differential expression analysis allowed for the identification of the clusters as astrocytes, hippocampal neurons, oligodendrocytes, or smooth muscle cells. Using transcriptomic and immunofluorescent imaging of AD mice brain tissue, MUSE was able to identify DEGs in individual clusters [172]. It was found that known AD-related genes *RANBP9* (downregulated in hypothalamus), *IGF1* (upregulated in cortex), and *SORL1* (upregulated in hypothalamus; downregulated in cortex) were differentially expressed [172]. This approach has revealed regional, temporal, and biological differences reflecting AD progression in a mouse model [172].

Spatial transcriptomics and proteomics have substantially advanced our ability to detect the heterogeneity of RNA and protein expression in tissues, although characterizing whole-transcriptome data of individual cells in space remains a challenge [173]. Integrating spatial transcriptomics with scRNA-seq and snRNA-seq techniques has been on the rise with hopes of resolving the limitations that spatial transcriptomics currently pose by gaining a deeper understanding of cell-cell communication within healthy and disease tissues and the roles certain cell subpopulations have in maintaining homeostasis and disease pathogenesis [[174], [175], [176], [177]]. With the potential to incorporate other types of spatial omics datasets as they become available to advance towards spatial multi-omics [178], we will be a step closer to elucidate the detailed tissue organization, cell regulation, and cellular communication at an unprecedented scale.

## 3 Towards integrative multi-omics and systems bioinformatics to reconstruct brain profiles

Neuroscience is being propelled into the big data era with an exponential increase in the amount of information generated which demands for better data organization, improved pipeline frameworks, and rapid turnover for analysis and interpretation to turn this information into valuable biological insights [179]. A systems bioinformatics based data mining approach enables the integration of a spectrum of multi-omics information ranging from epigenetics/epigenomics, transcriptomics, and proteomics to metabolomics and lipidomics by a combination of data-driven bioinformatics (top-down approach) and systems biology (bottom-up approach) [14]. It has also been proposed that establishing multiple networks representing information obtained from each type of omics dataset and integrating them in a layered network that exchanges information within and between layers could enable the comprehensive systems bioinformatics analysis (Fig. 1) [14]. One of the main challenges of integrative approaches is related to increased dimensionality, due to increased complexity of the omics dataset in the biological system. Experimentally, several neurodegenerative focused studies have started to incorporate a multi-omics approach in their analyses [[180], [181], [182], [183], [184]].

**Fig. 2:**
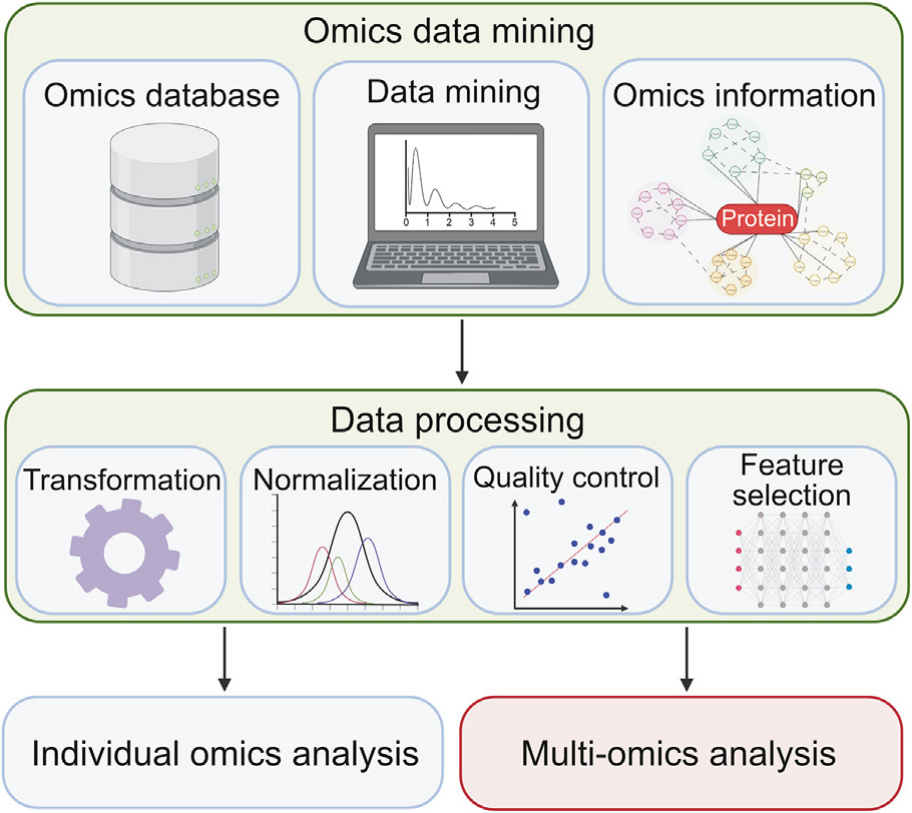
Omics data mining and data processing for individual and multi-omics analysis. Omics data mining involves the exploration of various omics databases to search for suitable datasets to be used, mining of the data, and obtaining the pre-processed omics information. The data must then be transformed and processed through normalization, quality control, and feature selection to extract interpretable information. The figure was created with BioRender.com.

**Fig. 3:**
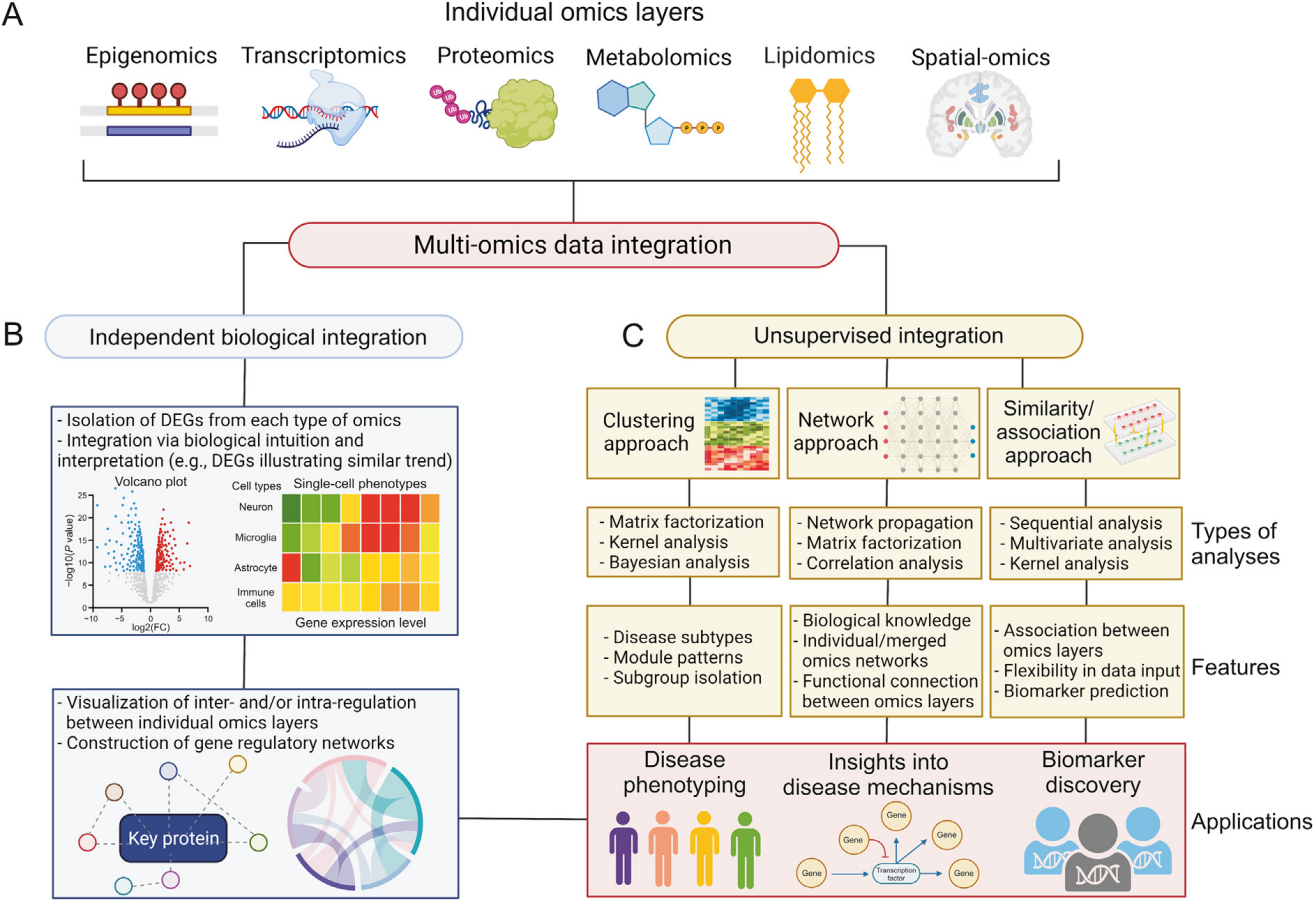
Multi-omics data integration of individual omics layers. (A) Multi-omics data integration is typically performed through two main approaches, namely the independent biological integration and the unsupervised integration. (B) The independent biological integration approach involves the isolation of differentially expressed genes (DEGs) from each omics layer followed by integration via biological intuition and interpretation as well as network visualization and construction. (C) The unsupervised integration involves three main categories of analysis which includes clustering, network, and similarity/association approaches. Each approach makes use of various statistical, network, correlation, and sequential analyses as well as incorporates different features for the integration. Both independent biological integration and the unsupervised integration can provide insights into disease phenotyping, disease mechanisms, and biomarker discovery. The figure was created with BioRender.com.

In both individual and multi-omics analyses, after data mining of the omics datasets, the data must be transformed and processed through normalization, quality control, and feature selection to extract interpretable information (Fig. 2). Normalization is typically applied to most omics layers to remove bias, large variation, and outlier or incorrect reads in order to make better comparison between different datasets or omics layers [185]. Quality control should be applied to all omics layers such as through quantifying GC content in RNA-seq and removing duplicated and fragmented reads for sequence alignment to be performed [186]. Feature selection is commonly conducted to reduce the dimensionality and redundancy of the high-throughput data, and discriminate desired features contained within the data [187,188]. The goal of the data processing is to reduce dimensionality, bias, and variation of the mined data in order to ensure robustness and efficiency of analysis, especially prior to multi-omics integration. Next, we describe two multi-omics integration approaches in data mining, namely independent biological integration and unsupervised integration, to combine individual layers of omics data for an integrative multi-omics analysis (Fig. 3A).

### 3.1 Independent biological integration

In independent biological integration, different types of omics datasets are typically analyzed by isolating the DEGs at the individual layers first and then compiled for integrated analysis. Integration is then performed by biological intuition such as comparing expression levels of genes and proteins to understand the translational regulation [189]. Integration can also be done through computational based or web-based tools that integrate expression levels of various types of omics data for biological interpretation, functional annotations, gene set enrichment, and multiscale network-based visualization to understand the interplay of regulation at different levels of gene expression [190,191] (Fig. 3B). Some of the computational tools include Kyoto Encyclopedia of Genes and Genomes (KEGG) [192,193], GO [194,195], DAVID [196], PANTHER [197,198], GSEA [199], and IPA [200] for pathway and gene enrichment analyses. The PPIs in functional modules and their interactions with each other in cellular networks can be determined by STRING [201], Cytoscape [202], NPA [203], and SPIA [204]. It is important to note that there might be variability between these computational tools, and it is advisable to use these computational tools in combination to define the most relevant and highly reproducible pathways and networks associated with certain disease states. To ensure robustness and accuracy of results, there must be consistency in parameters used for statistical analysis in each individual omics layer.

Other web-based tools such as MetExplore [205], 3Omics [206], and PaintOmics [207] have started to layout the potential of multi-omics analysis and visualization based on the independent biological integration approach [208]. The MetExplore program enables the visualization and interpretation of omics data from multiple molecular layers with inputs from different omics data followed by providing an interpretation of genome-scale metabolic networks and how various types of omics data modulate metabolic processes [205]. While the 3Omics program builds correlation networks, and enables phenotyping based on data from different omics layers [206], the PaintOmics programs allow for network visualization and accepts a wide variety of omics data [207]. The PaintOmics program additionally allows for pathway and enrichment analyses based on KEGG, Reactome, and MapMan databases. Using independent biological integration, omics datasets can be analyzed from multiple samples and do not need to be isolated from the same experiment. More complex forms of independent biological integration include “horizontal” (with features as anchors), “vertical” (with cellular information as anchors), and “diagonal” (no anchor) integration that are used for data mining of multi-modal single-cell datasets with anchors aligning different types of omics data [209]. Independent biological integration is currently being used as the primary method of omics integration in data mining studies due to ease of implementation without the need for highly technical computational competencies.

With the emergence of databases making multiple types of omics data publicly accessible, proof-of-concept integration of multi-omics datasets has been illustrated in data mining using the independent biological integration approach. A data mining study utilizing transcriptomic and proteomic datasets found significant DEGs in the human spinal cord of MS patients and the implications of these DEGs on biological processes involved in the disease progression of MS [189]. Specifically, they found that *HOXA5* was significantly upregulated in MS patients through individual transcriptomic (ARCHS4 database [56]) and proteomic analysis (BioGrid [210]) and that *HOXA5* was found to promote the transforming growth factor (TGF)-beta pathway [189]. A previous study has shown that in the spinal cord of MS patients there are large areas of demyelination characterized by a unique TGFB1 genomic signature [189]. This study proposes that the overexpression of *HOXA5* in the spinal cord may promote the progression of TGFB1-mediated gliosis in MS patients [189].

Multi-omics integration has also been reported to be conducted with the help of the MetExplore program to process genome-wide association studies (GWAS), transcriptomics, and proteomics datasets obtained from several databases (GWAS catalog [34], GEO [38], and PRIDE [92], respectively) to extract differentially expressed multi-omics elements [190]. This study identified 203 differentially expressed transcripts, 164 differentially expressed proteins, and 58 differentially expressed GWAS-derived mouse orthologs associated with significantly enriched metabolic biological processes [190]. Additionally, lipid metabolic pathways were significantly upregulated across the multi-omics datasets, with microglia and astrocytes expressing significant enrichment in the lipid-predominant AD-metabolic transcriptome [190]. This study brings attention to the significance of dysregulated lipid metabolism in AD, and the importance and usefulness of using multi-omics analysis to better understand AD pathogenesis from a systems bioinformatics approach, with experimental metabolomics/lipidomics validation in a mouse model and in the blood plasma of AD patients, respectively [190]. Although there are several advantages using the independent biological integration approach, it is worth noting that different studies that make use of this method may subject to different biological intuitions in the integration process, leading to non-standardized analysis and less consistent results.

### 3.2 Unsupervised integration

For unsupervised integration, multi-omics data from the different molecular layers are often concatenated together into a single matrix for analysis via ensemble dimension reduction [211,212]. Other approaches, such as model-ensemble, each omics layer is analyzed independently to obtain the respective matrix and the matrices from all omics layers are then inputted into the unsupervised algorithm and fused to build an integrated analysis. There are three main categories of unsupervised integration of multi-omics data, namely clustering-based, network-based, and similarity/association-based approaches (Fig. 3C). First, the clustering-based approach is primarily based on statistical calculations, making use of matrix factorization, kernel, and Bayesian analyses. Using matrix factorization analysis, the non-negative matrix factorization (NMF) method is most commonly utilized for high-dimensionality datasets and restricts their entries to non-negative values, allowing for easier interpretation of results [213]. Extensions of NMF include integrative NMF and joint NMF which account for the identification of heterogeneity and homogeneity in datasets respectively during the integration process [214,215]. Kernel analysis captures the degrees of similarity of the input data which are contained within the kernel matrix. This analysis is dimension-free and does not depend on the total number of features in the datasets. In the Bayesian analysis or Bayesian consensus clustering, a probability model such as the Dirichlet process mixture model, is used to model source-specific features as well as an overall clustering accounting for multiple data sources in different omics layers [216]. Clustering-based methods are suitable for identifying disease subtypes and module patterns, as well as isolating subgroups, samples, or features that have similar biological function.

The network-based unsupervised integration relies on biological knowledge databases for information on functional relationships between omics layers in addition to statistical analysis and calculation, and are heavily used to identify functional relationships between omics layers [212]. Network propagation analysis tracks the flow of each node and amplifies the signals based on the assumption that genes underlying similar phenotypes interact with one another through known information such as the association with a biological process [217]. Individual networks are then fused together into a similarity network using a nonlinear fusion approach which is based on message-passage theory [218]. Similar to the clustering-based method of analysis, network-based methods also utilize matrix factorization statistical approaches. On the other hand, correlation analysis is based on the correlation between a node, such as a gene and an outcome, with the significance of a node determined by the correlation coefficient or a regression-based significance [219].

Finally, the similarity/association-based unsupervised integration approach enables the identification of the marginal associations and correlations between various omics layers. Sequential analysis is an example, where statistical tests and models are applied to narrow down the list of features in one omics layer based on their relationship with features in other omics layers [212]. Multivariate analyses including canonical correlation analysis (CCA) and co-inertia analysis (CIA) are useful methods due to its flexibility in accepting multiple matrices as input data. While CCA can be applied for feature selection and classification in high-dimensional multi-omics datasets, CIA is used to find the low-dimensional components and aims to distinguish sources of variation in multi-omics datasets [220,221]. Similarity/association-based methods can also utilize kernel statistical analysis similar to clustering-based methods. Similarity/association-based unsupervised integration approach enables biomarker prediction, associations between omics layers (e.g., genotypes based on gene expression) and flexibility in accepted data (e.g., multiple matrices).

There are also some commonly used programs that can be utilized to facilitate unsupervised integration such as iCluster programs [222,223], JIVE [224], CNAmet [225], and PARADIGM [226]. Briefly, the iCluster programs and JIVE are all matrix-factorization-based clustering methods used for disease subtyping. The iCluster programs create flexible models based on the associations between omics layers and determines the variance-covariance within omics layers in a single framework, all while simultaneously reducing dimensionality of each omics layer [222]. The JIVE program is an extension of principal component analysis (PCA) and calculates the amount of joint variation between omics layers, reduces dimensionality, and enables visual exploration of joint and individual structures such as patterns of biological relationships between omics layers [224]. The CNAmet program is a similarity/associated-based sequential analysis tool that integrates high-throughput copy number, DNA methylation, and gene expression data that is used for biomarker prediction [225]. The PARADIGM program is a Bayesian-based network integration tool used for disease mechanistic studies and subtyping. It integrates multi-omics data to infer the modulation of genetic pathways based on established knowledge of the given pathways [226].

Unsupervised integration of multi-omics datasets has been mainly used in primary research, including AD, PD and ALS studies [4,12,227,228]. Recently, this approach has been adopted by neurodegenerative disease related data mining studies. For example, a study utilizes a combination of proteomics and lipidomics datasets collected from the blood of 586 AD patients and controls from other studies [229,230] for multi-omics analysis using the unsupervised integration approach [231]. Network analysis of the individual omics datasets was conducted using the Weighted Gene Correlation Network Analysis (WGCNA) which can also be applied to proteomics and lipidomics data [232,233]. Data processing which includes normalization, imputation of missing values, and PCA were performed before creating the weighted co-expression networks via hierarchically clustering and module assignments using a dynamic tree-cutting algorithm. To integrate the protein and lipid modules and analyze the associations between AD-associated modules, Pearson's correlation coefficient was utilized [231]. GO enrichment analysis was then used to analyze the biological processes as well as molecular and cellular functions of the protein modules associated with AD phenotypes. The study identified lipid modules involved in immune response and lipid metabolism as well as protein modules involved in increased cytokine production, humoral immune responses, and neutrophil-mediated immunity, all of which were highly correlated with AD risk loci [231]. This study is a good example of an unsupervised multi-omics integration approach via data mining which exemplifies network-based approaches for isolating protein and lipid modules that are highly associated with established AD risk loci. Another example related to brain disease is a glioblastoma study making use of multi-omics datasets obtained from patients to develop a network-correlation-based method called Lemon-Tree for biomarker discovery [234]. This study demonstrated that the Lemon-Tree algorithm successfully identifies known oncogenes and tumor suppressors as master regulators in the inferred module network, utilizing somatic copy number and expression data. Lemon tree allows the addition of other omics features such as miRNA and DNA methylation to be added to the model, and for GO enrichment analysis of the modules.

Currently, the largest challenges with overcoming interoperability of omics data is the lack of a standardized framework or pipeline, to enable various types of omics data to be seamlessly integrated and analyzed [208,212]. Systems bioinformatics extracts disease relevant information from multiple levels of the omics spectrum and integrates them in a layered and interactive network that exchanges systemic knowledge towards developing a comprehensive brain profile [[235], [236], [237]]. Although the process of optimizing the derived results is certainly required to obtain reliable biological information, this approach is theoretically applicable to the whole omics spectrum to work towards achieving systems bioinformatics in translational neuroscience. Our proposed concept focuses on a broad idea of obtaining ultimate research goals of reconstructing human brain systems with direct healthcare relevance [238], rather than a detailed in-depth analysis pipeline or algorithm development to analyze specific datasets [[239], [240], [241]]. We note here that highly technical methodologies, mathematical algorithms, and information theory are necessary to understand the omics derived networks.

## 4 Data mining and experimental validation in translational neuroscience

**Fig. 4:**
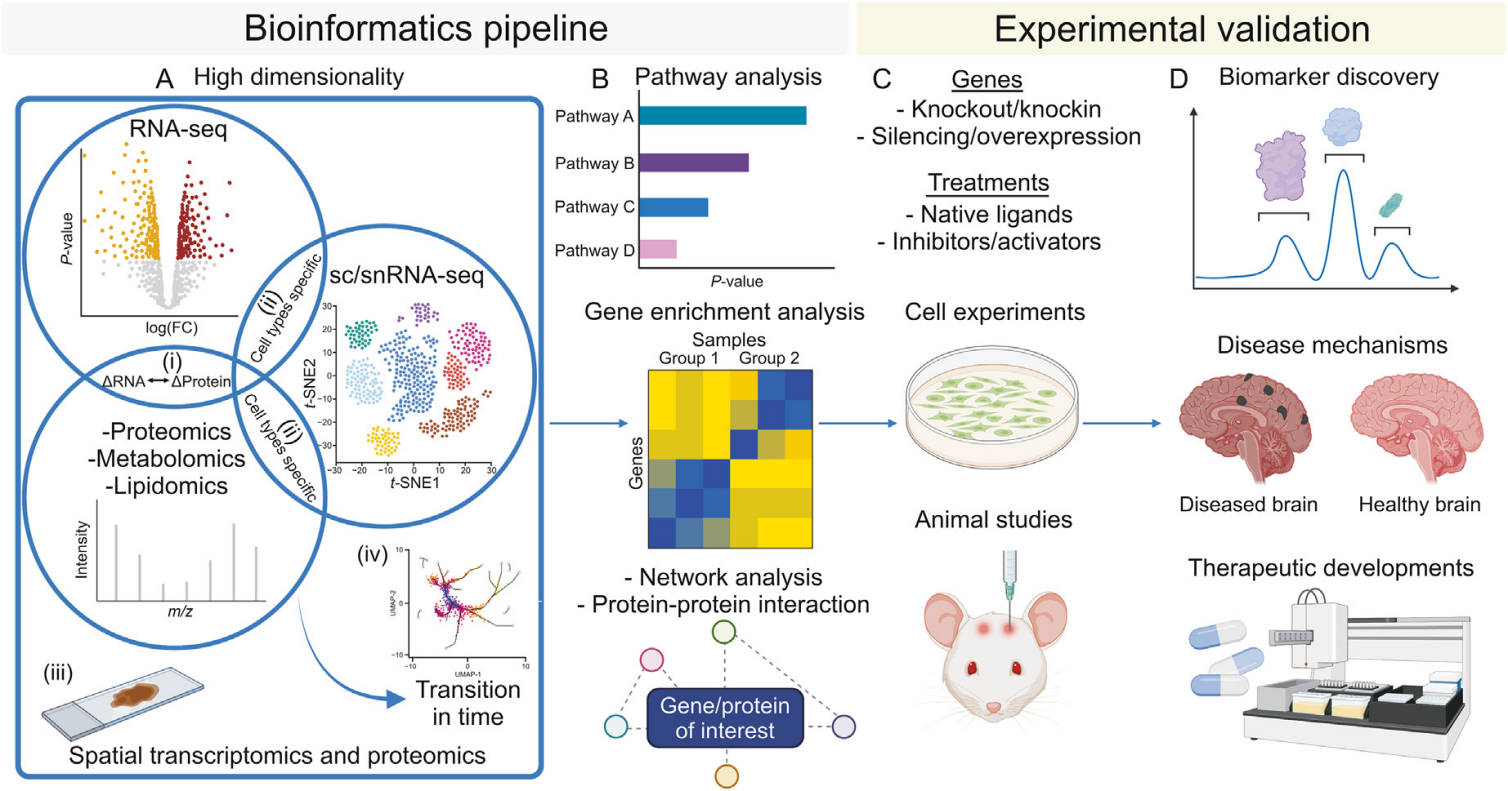
Proposed bioinformatics pipeline with experimental validation for translational neuroscience applications. (A) The high dimensional omics analysis includes (i) the overlap between transcriptomics and proteomics/metabolomics/lipidomics datasets which identifies the differentially expressed genes (DEGs) that are altered across all omics layers; (ii) the overlap with single-cell/single-nucleus RNA-sequencing (sc/snRNA-seq) indicates the identification of DEGs in different cell types or cell subpopulations; (iii) the overlap with spatial analysis enables the understanding of the location of the DEGs in the respective brain regions; and (iv) the overlap with data with time element will provide further information on how the DEGs and the corresponding phenotypes could change over time. (B) Key cluster of DEGs can be further analyzed by pathway analysis, gene-enrichment analysis, network analysis and/or protein-protein interaction analysis, among many other analyses that can be performed. (C) The key DEGs should be validated by cell experiments or animal studies with strategies such as gene knockout or knockin, gene silencing or overexpression, or treatments with native ligands or protein inhibitors/activators. (D) Changes in gene/protein levels or alteration in protein activities associated with certain disease phenotypes may provide insights to disease mechanisms, as well as the identification of potential biomarkers or advancement of potential therapeutic developments. The figure was created with BioRender.com.

Data mining approaches have advantages of being high throughput and low cost as compared to traditional low throughput experimental techniques in revealing the underlying pathogenic mechanisms of complex neurodegenerative diseases. However, prediction results arising from data mining remain theoretical and require validation with experimental evidence [242]. Here, we recommend an integrative neuroscience approach that synergizes systems bioinformatics and experimental analysis to yield opportunities for translational applications including biomarker discovery, therapeutic development, and insights into disease mechanisms (Fig. 4).

In the previous section, we have described multi-omics approaches to integrate different individual omics data together for a more holistic interpretation of the results. Besides omics data, high dimensionality analysis should include cell type, spatial location, and time trajectory (Fig. 4A). The knowledge of the biological functions and network systems in the brain such as the key pathways, genes, and PPI involved are important to piece all information together to provide interpretable biological findings (Fig. 4B). Under the context of multi-omics and systems bioinformatics analysis, one would expect consistent or correlated alterations in different omics layers, be it localized information or circulating information, to enable the reconstruction of the brain profile and understanding of the brain physiology. Nuanced analysis might be encountered which requires further optimization in data analysis and confirmation through experimental validation. Experimental validation bridges the gap between bioinformatics analysis and translational neuroscience and increases the credibility of results, especially for novel discoveries. For example, it is important to test how the overall biological system of cells and/or mice would react to alterations in certain key protein achieved by either overexpression/knockout or treatment of protein ligands/modulators (Fig. 4C). It is also important to examine whether these alterations have any adverse consequences on the model organisms tested such as toxicity. Generally, a protein plays a key role in disease mechanism if the change in the protein level or function correlates with disease pathogenesis or progression. Therapeutic discovery can be achieved through screening of small molecules or antisense oligonucleotide that can modulate the protein function. A biomarker is typically established if alterations in certain key proteins that are associated with disease pathogenesis can be detected in blood or CSF prior to disease progression (Fig. 4D).

Overall, it is essential for computational scientists, experimental biologists, and neuroscientists to communicate and exchange the intricacies of their individual methods to enable evaluation and validation of results with appropriate interpretation in a balanced manner [243]. Integrative neuroscience, combining both systems bioinformatics and experimental biology analysis, is a multidisciplinary science that can provide a new approach for biomarker discovery, therapeutic development, and elucidation of pathogenic mechanisms of neurodegenerative diseases [239].

## 5 Summary and future perspectives

Bioinformatics is becoming increasingly essential for the organization and management of data in modern biology and is a comprehensive field that harnesses methods in computer science and experimental biology. Bioinformatics is redefining modern science in ways that were not possible in the past with the ability to combine datasets from multiple experiments and different samples for large analysis has never been as accessible and accurate as today, yielding ever-growing applications of bioinformatic analyses [244]. Systems bioinformatics is a rising concept which makes use of network-based computational analysis to increase the precision of mechanistic understanding of disease pathogenesis as well as development of new diagnostics and therapeutics [14].

This review accentuates the importance of understanding the applications and boundaries of various data mining approaches of omics datasets and computational methods towards multi-omics analysis. The integration of multi-omics analysis, systems bioinformatics, and experimental validation provides insights into disease mechanisms and opens avenues for translational neuroscience applications as well as advancing towards early diagnosis [245], precision phenotyping of diseases [246] and personalized medicine [247]. With a resolution of exactly understanding the molecular level changes of a certain proteins in a specific cell-type as well as its detailed location and trajectory in time, it is theoretically possible to target the exact cells with the pathological features and provide treatments, although this will require highly specific targeting strategies to be developed.

A number of limitations remain for systems bioinformatics and experimental analysis in translational neuroscience before it can be fully implementable [248,249]. A major challenge lies in validating the reconstructed molecular networks with real biological observations as there is a lack of ground truth and technical capabilities to recapitulate the layered networks in the brain [14]. It has been suggested that an initial step would be to compare reconstructed networks with benchmarking datasets with known biological information such as from existing curated databases and simulated datasets that mimic real data [250]. In addition, similar to the current limitation in data mining where there is no streamlined pipeline for bioinformatics analysis, there is also variability in the network reconstruction approaches, leading to inadequate network selection and inconsistent results between different studies [251]. Hence, there is a need for computational approaches to screen results from different methods of analyses to provide a consensus analysis that will maximize the information content, although this remains to be developed.

Heterogeneity of neurodegenerative diseases within individuals is caused by multiple complex factors and is impeding development of effective treatments, leading to the notion of personalized medicine. Systems bioinformatics methodologies enable the collection of invaluable knowledge gathered from the different aspects of a disease condition and provide revolutionary approaches and tools to clinicians to demystify the complex nature of these diseases. Although the full implementation of the systems bioinformatics approach to reconstruct the human brain profile might seem ambitious at the current stage, its current application might be complementary with the use of existing computational and translational neuroscience methods. The practical integration of systems bioinformatics and experimental analysis in translational neuroscience is likely to have a major impact and significant breakthroughs in detection and diagnostics of neurological disorders and neuroscience targeted drug discovery in the future [252], and may be applicable to studying other diseases in general.

## CRediT author statement

**Lance M. O’Connor:** Data curation, Formal analysis, Investigation, Methodology, Visualization, Writing - Original draft preparation; **Blake A. O’Connor:** Data curation, Writing - Reviewing and Editing; **Su Bin Lim:** Writing - Reviewing and Editing; **Jialiu Zeng:** Funding acquisition, Validation, Writing - Reviewing and Editing; **Chih Hung Lo:** Conceptualization, Funding acquisition, Investigation, Project administration, Supervision, Validation, Visualization, Writing - Original draft preparation.

## Declaration of competing interest

The authors declare that there are no conflicts of interest.
